# Constructing Direct Z‐Scheme Heterostructure by Enwrapping ZnIn_2_S_4_ on CdS Hollow Cube for Efficient Photocatalytic H_2_ Generation

**DOI:** 10.1002/advs.202201773

**Published:** 2022-06-24

**Authors:** Chuan‐Qi Li, Xin Du, Shan Jiang, Yan Liu, Zhu‐Lin Niu, Zhong‐Yi Liu, Sha‐Sha Yi, Xin‐Zheng Yue

**Affiliations:** ^1^ College of Chemistry Zhengzhou University Zhengzhou 450001 China; ^2^ Henan Institutes of Advanced Technology Zhengzhou University Zhengzhou 450003 China; ^3^ School of Materials Science and Engineering Zhengzhou University Zhengzhou 450001 China

**Keywords:** density functional theory, direct Z‐scheme junction, hollow CdS cube, photocatalytic H_2_ generation, ZnIn_2_S_4_/CdS heterostructure

## Abstract

Rational design hybrid nanostructure photocatalysts with efficient charge separation and transfer, and good solar light harvesting ability have critical significance for achieving high solar‐to‐chemical conversion efficiency. Here a highly active and stable composite photocatalyst is reported by integrating ultrathin ZnIn_2_S_4_ nanosheets on surface of hollow CdS cube to form the cube‐in‐cube structure. Experimental results combined with density functional theory calculations confirm that the Z‐scheme ZnIn_2_S_4_/CdS heterojunction is formed, which highly boosts the charge separation and transfer under the local‐electric‐field at semiconductor/semiconductor interface, and thus prolongs their lifetimes. Moreover, such a structure affords the highly enhanced light‐harvesting property. The optimized ZnIn_2_S_4_/CdS nanohybrids exhibit superior H_2_ generation rate under visible‐light irradiation (*λ* ≥ 420 nm) with excellent photochemical stability during 20 h continuous operation.

## Graphical abstract

The stable composite photocatalyst integrated by ultrathin ZnIn_2_S_4_ nanosheets on surface of hollow CdS exhibits outstanding visible‐light‐driven photocatalytic H_2_ generation activity, due to that: i) the Z‐scheme charge transfer route facilitates charge separation, prolongs carrier lifetimes, and remains strong electron reduction capacity; ii) the unique cube‐in‐cube structure brings abundant active sites, improves light harvesting ability, shortens charge transfer distance.

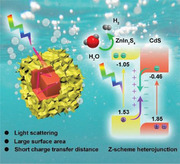

## 1 Introduction

With the rapid development of industrial society, developing promising alternatives such as hydrogen (H_2_) with inexhaustible, clean, and renewable features to fossil fuels becomes extremely essential.^[^ ^1^, ^2^, ^3^ ^]^ Photocatalytic H_2_ generation from water splitting is one of the most promising techniques which depend on the efficient and robust photocatalysts with wide light absorption range, high charge carrier separation efficiency, stable recycle performance as well as strong redox ability. Unfortunately, it is virtually impossible for a single catalyst to possess all above mentioned features and perform photocatalytic activity to a satisfying level.^[^ ^4^ ^]^ Among the being‐studied strategies for hybrid construction, heterojunction draws public attention because the photogenerated charge carriers can timely separate and transfer to participate in the reaction under the effect of built‐in electric field. Especially, the building of Z‐scheme heterostructures by coupling complementary semiconductors has been commonly focused. In such a structure, the existing of internal electric field boosts the CB (conduction band)‐electrons of one semiconductor directionally transfer to the valence band (VB) of another one, thus the strong redox ability of electrons/holes can be achieved and retained simultaneously on different active sites independently.^[^ ^5^, ^6^ ^]^

Metal chalcogenide based photocatalysts show favorable visible‐light response ability, always being as the potential materials for achieving high H_2_ generation activity. CdS, as a representative material, possesses suitable band gap of 2.42 eV and CB potential of ‒0.43 eV,^[^ ^7^ ^]^ which holds strong reduction capacity for H_2_ generation from water splitting. However, the photocatalytic activity of pristine CdS is highly limited by the serious recombination of photogenerated electrons and holes.^[^ ^8^ ^]^ To address this issue, Nasir and co‐workers employed Co and Ni co‐catalysts to realize the efficient charge separation over CdS nanorods.^[^ ^9^, ^10^ ^]^ Recently, CdS‐based Z‐scheme heterojunctions such as g‐C_3_N_4_/CdS, TiO_2_/CdS, and CoS*_x_*/CdS have been constructed for achieving excellent H_2_ generation performances.^[^ ^11^, ^12^, ^13^ ^]^ To further pursue the ultimate photocatalytic activity, incorporating a semiconductor with more negative CB potential where electrons have much stronger reduction ability on CdS to form direct Z‐scheme heterojunction is urgently. By virtue of the appropriate CB and VB potentials, outstanding optical properties, and easy‐grow nature, ternary metal chalcogenide of ZnIn_2_S_4_ is an attractive material to combine with CdS for achieving Z‐scheme heterojunction.^[^ ^14^, ^15^, ^16^ ^]^ Meanwhile, the ideal shared sulfur atom between two semiconductors further promotes the intermolecular interactions for efficient charge transfer.^[^ ^17^ ^]^

Furthermore, engineering a unique functional photocatalyst with unique nanostructures can usually afford the material with preferable optical property and photophysical character, thus boosting the catalytic activity. Hollow‐structured materials have attracted much research interest by virtue of the following features:^[^ ^18^, ^19^, ^20^ ^]^ i) the rich active sites for redox reactions; ii) the shortened distance to transfer charge carriers; iii) the highly enhanced light absorption and utilization induced by light scattering and reflecting. As reported by Lou and co‐workers,^[^ ^21^ ^]^ the constructed hierarchical Co_9_S_8_@ZnIn_2_S_4_ photocatalyst by growing ZnIn_2_S_4_ nanosheets on the surface of Co_9_S_8_ dodecahedral cages afforded large surface area with abundant reactive sites, and the ability to promote the separation and transfer of photogenerated charge carriers.

Based on the above considerations, we synthesize the hierarchical ZnIn_2_S_4_/CdS heterostructures by in situ growing ultrathin nanosheets of ZnIn_2_S_4_ on outer surface of CdS hollow cubes. Such a unique structure not only provides abundant reactive sites for H_2_ generation, increases light utilization through enhanced scattering and reflection, but also shortens the distance for charge transport. Density functional theory (DFT) calculations revealed the Z‐scheme charge transfer route over ZnIn_2_S_4_/CdS heterojunctions, which promotes the separation of photogenerated electron‐hole pairs and maintains strong reduction ability of CB electrons for water splitting. As expected, the optimized ZnIn_2_S_4_/CdS photocatalyst exhibits remarkable cocatalyst‐free photocatalytic H_2_ generation rate under visible light irradiation and long‐term stability. This work provides a new idea for the synthesis of other semiconductor based promising nanostructures in future.

## 2 Results and Discussion

### 2.1 Characterization of Morphology and Structure

**Figure 1:**
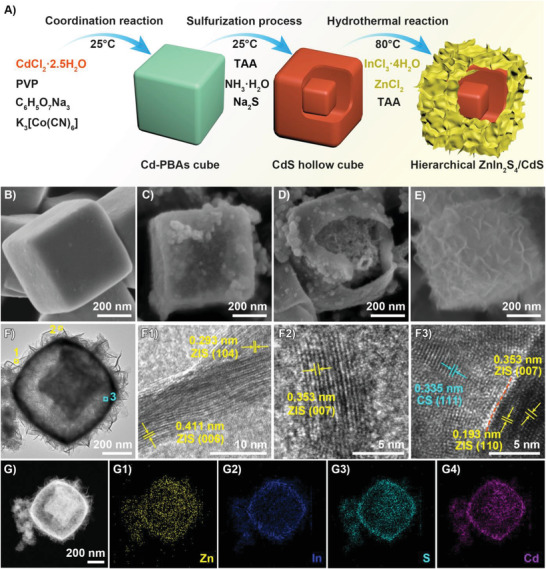
A) Schematic illustration of the synthesis procedure of hierarchical ZIS/CS. FIB‐SEM images of B) Cd‐PBAs cube, C,D) CdS hollow cube and E) 30% ZIS/CS. F) HRTEM images of 30% ZIS/CS. (F1–F3) are the enlarged views of the circled areas in (F). G) HAADF‐STEM image and EDX elemental mappings of G1) Zn, G2) In, G3) S, and G4) Cd over ZIS/CS.

The synthetic strategy for the hierarchical ZnIn_2_S_4_/CdS (ZIS/CS) hybrid photocatalyst includes three steps, as demonstrated in **Figure** **1**A. In the first step, Cd‐PBAs (Prussian blue analogs) precursor was synthesized by a mild coordination reaction at room temperature.^[^ ^19^ ^]^ Focused ion beam scanning electron microscope (FIB‐SEM) image reveals that Cd‐PBAs display a solid cube mophology with side length around 400 nm (Figure 1B). The second step is a sulfurization process at room temperature to convert Cd‐PBAs into CdS using thioacetamide (TAA) and Na_2_S as S precursors. It can be seen in Figure 1C,D that CdS displays the cubic hollow structure with an uniform size of 600 nm, revealing the sulfurization process had no obvious influence on the nanoparticle size. The third step features a hydrothermal reaction at 80 °C to uniformly grow ZIS nanosheets on the whole surface of hollow CdS and achieves the cube‐in‐cube nanostructure, as confirmed by the FIB‐SEM image shown in Figure 1E. As for bare ZIS, it presents flower‐like spherical nanostructure assembled by ZnIn_2_S_4_ nanosheets (Figure S1, Supporting Information).

Transmission electron microscopy (TEM) observation further confirms the cube‐in‐cube structure of ZIS/CS, and the ZIS outer shell is composed of ultrathin nanosheets (Figure 1F). This unique structure is so attractive compared with previously reported systems.^[^ ^22^, ^23^ ^]^ The lattice fringes of the ZIS nanosheets were observed through high‐resolution TEM (HRTEM, Figure 1F1,F2), in which the interplanar spacings of 0.411, 0.353, and 0.293 nm can be indexed to the (006), (007), and (104) planes of ZnIn_2_S_4_, respectively, in consistent with the X‐ray diffraction (XRD) result (*vide‐infra*). Furthermore, the highly crystalline heterointerface in the ZIS/CS hybrid can be clearly identified by HRTEM image in Figure 1F3, in which the 0.353 and 0.193 nm can be assigned to (007) and (110) interplanar spaces of ZIS, and the 0.335 nm belongs to (111) plane of CS. To further gain insight into the structure of the ZIS/CS hybrid photocatalyst, scanning TEM‐energy‐dispersive X‐ray (STEM‐EDX) characterization was conducted to obtain elemental composition and distribution information. In Figure 1G, elemental mapping images suggest that In (blue) and Zn (yellow) are limited to the connected nanosheets of the outer cube, whereas Cd (cyan) concentrates at the inside and outside of hollow cube, and S (purple) is uniformly distributed throughout ZIS/CS structure (Figure 1G). Further, EDX spectrum (Figure S2, Supporting Information) illustrates signals of Zn, In, Cd, and S elements, further demonstrating the composition of ZnIn_2_S_4_ and CdS in hybrid material.

**Figure 2:**
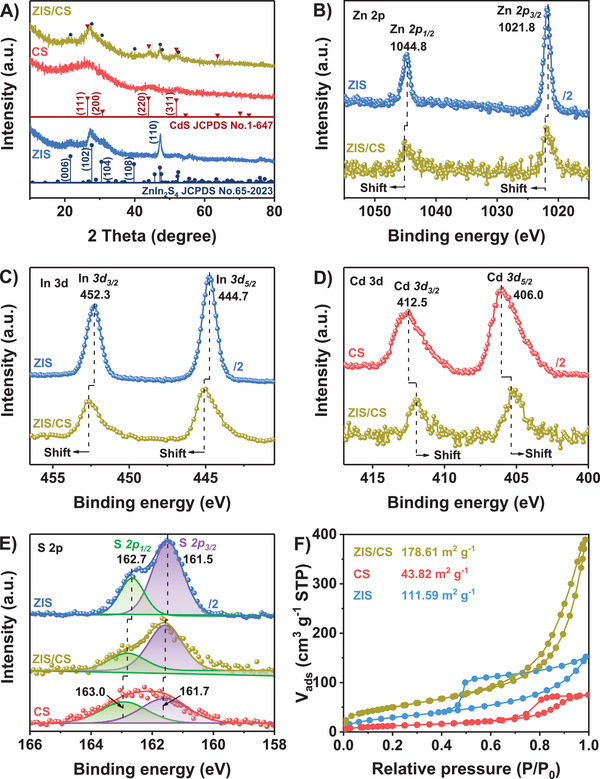
A) XRD patterns of ZIS, CS and ZIS/CS. B) Zn 2p and C) In 3d XPS spectra of ZIS and ZIS/CS. D) Cd 3d XPS spectra of CS and ZIS/CS. E) S 2p XPS spectra of ZIS, CS and ZIS/CS. F) N_2_ adsorption‐desorption isotherms of ZIS, CS, and ZIS/CS.

XRD measurements were employed to investigate the crystalline structure of the catalysts. For the CS and ZIS shown in **Figure** **2**A, all their diffraction peaks matched those of the cubic phase of CdS (JCPDS No. 1‐647)^[^ ^19^ ^]^ and hexagonal phase of ZnIn_2_S_4_ (JCPDS No. 65‐2023),^[^ ^24^ ^]^ respectively. For XRD pattern of ZIS/CS, not only peaks corresponding to CdS but also the ZnIn_2_S_4_ could be observed, respectively, suggesting the successful synthesis of heterojunction. Furthermore, the composition and chemical states of photocatalysts were analyzed based on the X‐ray photoelectron spectroscopy (XPS). Survey XPS spectra of CS and ZIS reveal the existence of Cd and S, and Zn, In and S elements, respectively, whereas elemental Zn, In, S, and Cd signals were detected over ZIS/CS (Figure S3, Supporting Information). Figure 2B,C show the high‐resolution XPS spectra of Zn 2p and In 3d for ZIS and ZIS/CS samples. For Zn 2p, two characteristic peaks at binding energies (BEs) of 1021.8 and 1044.8 eV are observed (Figure 2B), corresponding to Zn 2p_3/2_ and Zn 2p_1/2_ of Zn^2+^ in ZnIn_2_S_4_.^[^ ^25^ ^]^ The In 3d XPS spectrum of ZIS shows BE components at 444.7 (In 3d_5/2_) and 452.3 eV (In 3d_3/2_), respectively, characteristic of In^3+^ (Figure 2C). The Cd 3d XPS spectrum of CS reveals two strong peaks at BEs of 406.0 (Cd 3d_5/2_) and 412.5 eV (Cd 3d_3/2_), confirming the predominance of Cd^2+^ valence form (Figure 2D).^[^ ^4^ ^]^ Figure 2E displays the S 2p XPS regions of CS, ZIS, and ZIS/CS catalysts, which were deconvoluted into two components. For ZIS/CS, S 2p_3/2_ (161.6 eV) and S 2p_1/2_ (162.9 eV) peaks were observed, which verifies the S^2−^ states.^[^ ^26^ ^]^ Note that, the positive shifts of Zn 2p and In 3d XPS peaks are observed on ZIS/CS compared to ZIS, whereas a negative shift of Cd 3d region for ZIS/CS appears when compared to CS. Such shifted BEs in XPS results suggest that the electrons transfer from CS to ZIS,^[^ ^27^ ^]^ which can be confirmed by the formation of interfacial band bending that support the direct Z‐scheme mechanism.^[^ ^4^ ^]^

To obtain the specific surface areas and porous structure information, the N_2_ adsorption‐desorption isotherms were carried out through the Brunauer–Enmet–Teller method. Both CS and ZIS/CS show the typical type IV isotherms with obvious hysteresis loops, revealing their mesoporous character.^[^ ^28^ ^]^ For CS and ZIS, the isotherms exhibit H2b type hysteresis behavior with saturated adsorption platform and reflect the uniform distribution of pores. In contrast, the isotherms of ZIS/CS show a H3 type hysteresis behavior without obvious saturated adsorption platform, indicating the irregular pore structures. As compared to CS and ZIS, ZIS/CS shows much larger specific surface area. These features afford ZIS/CS with shortened route for charge transfer, abundant active sites for water reduction, and good light harvesting ability by multiple light reflection and scattering, thus remarkably boosting the H_2_ evolution reaction.

### 2.2 Highly Enhanced Photocatalytic H_2_ Generation Activity

**Figure 3:**
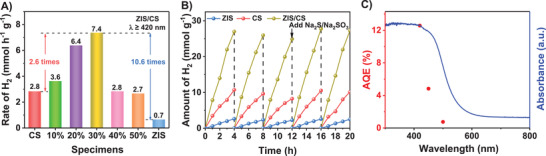
A) Comparison of photocatalytic H_2_ generation rates among CS, ZIS and *x*% ZIS/CS (*x* = 10, 20, 30, 40, and 50) under visible light irradiation (*λ* ≥ 420 nm). B) Recycling photocatalytic H_2_ generation tests of ZIS, CS, and ZIS/CS. C) UV–vis DRS (blue line) and wavelength‐dependent AQE (red symbols) of photocatalytic H_2_ generation over ZIS/CS.

Photocatalytic H_2_ generation from water splitting over ZIS, CdS and *x*% ZIS/CS (*x* = 10, 20, 30, 40 and 50) samples was evaluated in 0.35 m Na_2_S + 0.25 m Na_2_SO_3_ aqueous solution (pH 13.5) under visible light irradiation (*λ* ≥ 420 nm). As shown in **Figure** **3**A, the H_2_ generation rate for pristine ZIS nanoflowers is 0.7 mmol h^−1^ g^−1^, while for CS hollow cube, it is 2.8 mmol h^−1^ g^−1^. Notably, when ZIS was assembled on surface of CS, the H_2_ generation activity turned out to be dramatically boosted. The highest H_2_ generation rate was observed on 30% ZIS/CS, reaching a value of 7.4 mmol h^−1^ g^−1^, up to 2.6 and 10.6 times as high as those of CS and ZIS, respectively. Notably, increasing the loading amount of ZIS higher than 30% lowered the photocatalytic performance, which may due to the saturated heterojunction interface established between ZIS and CS as well as the aggregated ZIS nanoflows (Figure S5, Supporting Information) that influence the light harvesting. These photocatalytic results emphasize the pivotal role played by the designed ZIS/CS junction. Recycling tests on both CS and ZIS/CS were conducted to judge their stability performances during a long‐term reaction. It can be seen in Figure 3B that, compared with pristine CS, the reduction in H_2_ generation amount of ZIS/CS was relatively low during the first 12 h, suggesting the higher stability of our target heterojunction photocatalyst. After adding Na_2_S and Na_2_SO_3_ sacrificial agents, the amounts of H_2_ after 20 h illumination show no noticeable degradation for CS and ZIS/CS catalysts, indicating their stability, as confirmed by the XRD results before and after 20 h reaction (Figure S6, Supporting Information). The apparent quantum efficiency (AQE) of ZIS/CS at monochromatic light irradiation of 420 nm was calculated to be 12.6% (Figure 3C). In addition, similar wavelength‐dependent AQE values were observed following the optical absorbance of ZIS/CS.

### 2.3 Photo(Electro)Chemical Characterizations to Reveal the Superiority of ZIS/CS

**Figure 4:**
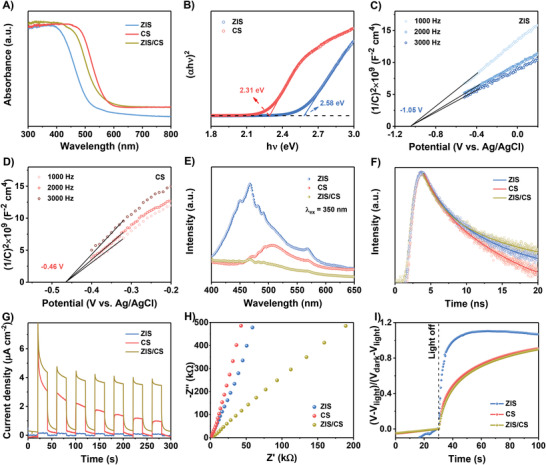
A) UV–vis DRS of ZIS, CS, and ZIS/CS. B) The corresponding (*α*h*ν*)^2^ versus h*ν* curves of ZIS and CS. M–S plots of C) ZIS and D) CS under frequencies of 1000, 2000, and 3000 Hz in dark condition. E) PL and F) TRPL spectra of ZIS, CS, and ZIS/CS. G) Transient *i–t* curves at 0 V versus Ag/AgCl, H) EIS Nyquist plots and I) normalized OCP decay plots of ZIS, CS, and ZIS/CS.

For a better understanding of the origin of the improved photocatalytic activity for ZIS/CS heterojunction, UV–vis DRS technique was first performed to study the light absorption property. As **Figure** **4**A shows, pristine CS and ZIS exhibit band‐edge absorption of 500 and 420 nm, respectively. After assembling ZIS nanosheets on CS hollow cube, a wider light absorption range and stronger absorption intensity are discovered compared to ZIS, indicating that ZIS/CS displays the efficient solar light harvesting in the visible light range, which is mainly due to the multiple reflections inside the cavity of the hollow structure. According to Kubelka–Munk method,^[^ ^29^ ^]^ the (*α*h*ν*)^2^ is plotted versus the photon energy (h*ν*), as illustrated in Figure 4B. By extrapolating the plot to (*α*h*ν*)^2^ = 0, the band gap (*E*_g_) values of ZIS and CS are estimated to be 2.58 and 2.31 eV, respectively. Furthermore, the conduction band (CB) levels of ZIS and CS are determined based on the Mott–Schottky (M–S) plots, which were recorded over frequencies of 1000, 2000, and 3000 Hz in dark, respectively. As Figure 4C,D shows, the M–S plots of ZIS and CS display positive slopes, indicating their n‐type semiconductor property.^[^ ^30^, ^31^ ^]^ According to the previous reports,^[^ ^17^, ^30^, ^32^ ^]^ the flat band potential (*E*_fb_) can be obtained by extrapolating the slope of the M–S plots to the *x*–axis (i.e., 1/*C* ^2^ =  0), which is located just below the bottom (0.2 V) of CB potential (*E*_CB_). Clearly, the *E*_fb_ of ZIS and CS are read to be −1.05 and −0.46 V versus Ag/AgCl, respectively, and their *E*_CB_ values are estimated to be −1.25 and −0.66 V versus Ag/AgCl, that is −1.05 and −0.46 V versus NHE (normal hydrogen electrode, *E*_NHE_ = *E*_Ag/AgCl_ + 0.197 V), respectively. Given their bandgap values (Figure 4B), the VB levels of ZIS and CS are calculated as 1.53 and 1.85 V versus NHE (Table S1, Supporting Information) according to *E*_VB_ = *E*_CB_ + *E*_g_,^[^ ^33^ ^]^ respectively.

To evaluate how the constructed heterojunction structure affects the photogenerated charge behaviors including the separation and dynamic information,^[^ ^34^ ^]^ the steady‐state photoluminescence (PL) and time‐resolved PL (TRPL) measurements of ZIS, CS, and ZIS/CS were carried out for comparison. As accepted, the signal intensity of PL spectrum is related to the recombination of photogenerated charge carriers. As displayed in Figure 4E, the PL signal intensity of ZIS/CS is obviously quenched compared to ZIS and CS, revealing the low recombination rate of photogenerated electrons and holes in ZIS/CS. In addition, in comparison to ZIS and CS, ZIS/CS shows an increased average PL lifetime (*τ*, Τable S2, Supporting Information), indicating that the decorating of ZnIn_2_S_4_ on CdS can effectively inhibit the recombination of charge carriers. Furthermore, the charge separation and transfer efficiencies were conducted by a series of photo(electro)chemical techniques, such as transient current–time (*i–t*) curves, electrochemical impedance spectroscopy (EIS), and open circuit potential (OCP) decay profiles. As demonstrated in Figure 4G, the generated photocurrent density of ZIS/CS was much higher than those of ZIS and CS, which can be ascribed to the fact that the heterojunction is formed between ZIS and CS that results in efficient separation of electron‐hole pairs under the action of interfacial electric field. In addition, a smaller arc radius of EIS spectrum was observed for ZIS/CS as compared to those of ZIS and CS (Figure 4H), demonstrating a more effective charge separation and faster interfacial charge transfer in the ZIS/CS composite. To further investigate how the build internal electric field affects the surface charge behaviors of ZIS/CS heterojunction, we interrogated the ZIS, CS, and ZIS/CS samples by OCP decay profiles, which is a useful technique for tracking surface recombination rate of photogenerated charge carriers.^[^ ^35^, ^36^ ^]^ Figure 4I displays the normalized OCP response followed by termination the irradiation. More interestingly, the OCP decay rate of ZIS/CS is slower than those of ZIS and CS, which confirms the low surface recombination rate of photogenerated electrons and holes. In addition, the carrier lifetime as a function of OCP derived from OCP decay profile was shown in Figure S7, Supporting Information, revealing that ZIS/CS hybrid displays an obvious longer charge carrier lifetime than those of ZIS and CS, in agreement with the TRPL observations (Figure 4F). These results indicate that the extended lifetime of charge carriers can be ascribed to the constructed heterojunction in ZIS/CS.

### 2.4 Photogenerated Charge Separation Behaviors

**Figure 5:**
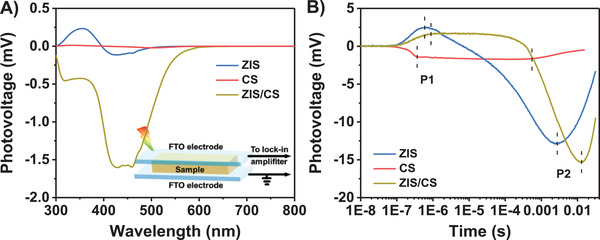
A) SPV and B) TPV spectra of ZIS, CS, and ZIS/CS. The inset displays the schematic setup for the SPV measurements.

To more directly investigate the charge behaviors including the separation, diffusion, and recombination at the surface and the interface from the point of photophysical mechanism, the surface photovoltage (SPV) and transient photovoltage (TPV) techniques were conducted over ZIS, CS, and ZIS/CS specimens.^[^ ^37^, ^38^ ^]^ For bare ZIS, an obvious positive signal emerges at ≈380 nm (**Figure** **5**A) related to the intrinsic transition, which means that photogenerated holes transfer to the surface of ZIS, while CS exhibits weak positive signal due to the rapid recombination of electrons and holes. In contrast, the negative and strong SPV signal is recorded on ZIS/CS, indicating that electron‐hole pairs are effectively separated and the electrons are migrated to the surface. The result shows that photogenerated electrons accumulate on ZIS/CS surface, which is favorable for photocatalytic H_2_ generation. The TPV spectra were conducted under wavelength of 355 nm laser. As shown in Figure 5B, the TPV responses of ZIS, CS, and ZIS/CS all reveal two peaks at a time shorter than 10^−6^ s (fast component, P1) and a time longer than 10^−5^ s (slow component, P2), which corresponds to the typical timescale of the surface photovoltage generated in the electric field and diffusion photovoltage, respectively. ZIS/CS exhibited the highest photovoltaic response in slow component, revealing the highest separation of photogenerated electron‐hole pairs. Moreover, ZIS/CS shows an obvious decay for TPV peak of P2 compared to those of CS and ZIS, indicative of the time retardation needed for separation and recombination process in hybrid catalyst. In other words, the possible charge transfer from CS to ZIS benefits the reduced recombination of charge carriers and thus prolongs their lifetimes. These results are good consistent with the observations of TRPL spectra and OCP decay profiles (Figure 4F,I).

### 2.5 In Situ Electron Paramagnetic Resonance Characterization

**Figure 6:**
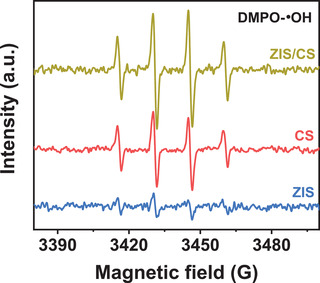
EPR signals of DMPO‐•OH spin adducts spectra of ZIS, CS, and ZIS/CS under visible light irradiation (*λ* ≥ 420 nm).

To better figure out the photocatalytic process of H_2_ generation over ZIS/CS, we performed the electron paramagnetic resonance (EPR) technique to in situ monitor the intermediates using 5,5‐dimethyl‐1‐pyrroline N‐oxide (DMPO) as a trapping regent of hydroxyl radicals (•OH). Under visible light irradiation, four characteristic signals of DMPO‐•OH with an intensity ratio of 1:2:2:1 were observed on EPR spectra of CS and ZIS/CS (**Figure** **6**), indicating that the hydroxyl radicals (•OH) were generated with the photocatalytic H_2_ generation reaction. However, as for the case of ZIS, the EPR signals of DMPO‐•OH adducts were hardly detected, meaning low amount of •OH is formed. Based on the reported redox potential of •OH/H_2_O (≈1.6–1.9 V versus NHE),^[^ ^39^, ^40^, ^41^ ^]^ we compared it with the *E*_VB_ of ZIS and CS (Table S1, Supporting Information), respectively. Apparently, only the VB level of CS owns the enough oxidation ability to achieve •OH, which means that VB‐holes of CS exist in the photocatalytic water reduction reaction. This is understandable when the CB‐electrons of CS can transfer to the VB of ZIS to construct a Z‐scheme photocatalysis system, allowing the high oxidation and reduction ability locate at CS and ZIS, respectively. The photocatalytic H_2_O_2_ generation results further prove the Z‐scheme mechanism of ZIS/CS heterostructure (Figure S8, Supporting Information). Hence, construction of a Z‐scheme heterojunction is the fundamental reason for enhanced H_2_ generation over ZIS/CS.

### 2.6 DFT Calculations

**Figure 7:**
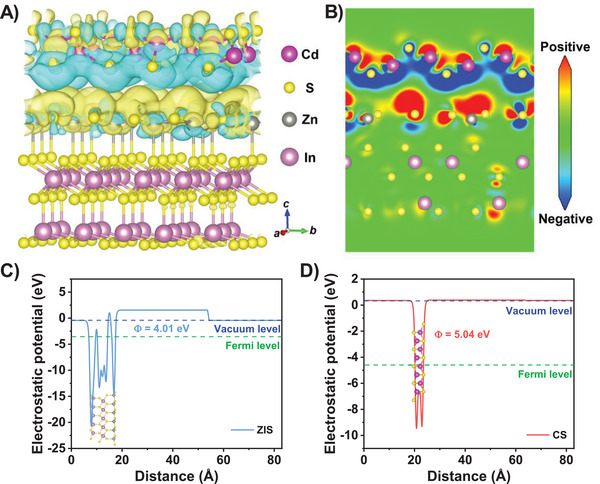
A) 3D model of the matched interface structure and charge density difference at ZIS(001)/CS(101) interface. B) The longitudinal section image of (A). The calculated work functions and corresponding structural model of C) (001) plane of ZIS and D) (101) plane of CS.

DFT calculations are implemented in the Vienna Ab initio Simulation Package (VASP) over the constructed heterojunction interfaces between (001) facet of ZIS and (101) facet of CS (Figure S9, Supporting Information). To illustrate the orbital hybridization information at the heterojunction interface, 3D charge density difference was calculated. As shown in **Figure** **7**A the electron clouds around S atoms in CS are scarce (blue region) while the electron clouds around Zn atoms in ZIS are significantly enriched (yellow region), which are linked together. This suggests that the ZIS side of the ZIS/CS heterojunction has the strong ability to enrich electrons. This situation can be seen more intuitively in the longitudinal section image of charge density difference (Figure 7B) which further indicates the transfer trend of electrons from CS to ZIS and thus proves the Z‐scheme charge transfer pathway in ZIS/CS heterojunction. Here the proposed Z‐scheme heterostructure for ZIS/CS is much more attractive than as‐reported staggered band alignment ones (such as type II heterojunction),^[^ ^22^, ^42^ ^]^ which enables the photogenerated charge carriers with high redox abilities.

Similar results can be obtained from the calculated electrostatic potential of ZIS(001)/CS(101) interface (Figure S10, Supporting Information). Clearly, CS exhibits electrophilic character while ZIS shows the nucleophilic one in the heterojunction interface, revealing that the holes and electrons are preferred to accumulate at CS and ZIS sides, respectively. Due to the presence of electrostatic potential, an upward electric field can be formed in the interface between ZIS and CS, which is favorable for the H_2_ generation.^[^ ^43^ ^]^ During the photocatalytic reaction, the CB electrons of CS flow to ZIS and recombine with the holes in VB position, in agreement with the charge density difference (Figure 7A,B). Figure 7C,D shows the calculated work functions (WFs) of ZIS (001) and CS (101) surfaces, that are 4.01 and 5.04 eV, respectively. This difference in their WFs will lead to the charges transfer from ZIS to CS to reach the Fermi level equilibrium and accordingly the interfacial band bending is formed. On the basis of the EPR, XPS, and DFT calculations, we confirmed that the Z‐scheme charge transfer pathway is existed in ZIS/CS heterojunction.

### 2.7 Photocatalytic Mechanism

**Figure 8:**
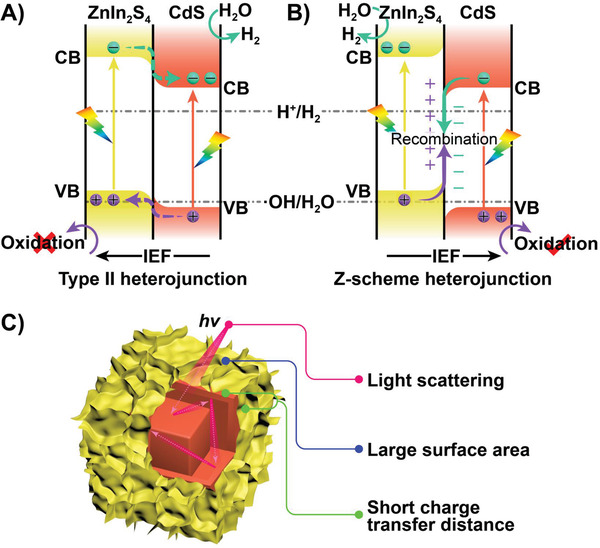
The energy level diagrams and charge transfer in A) type II and B) Z‐scheme ZnIn_2_S_4_/CdS heterostructure. C) Schematic illustration of some advantages of the hierarchical hollow cube‐in‐cube structure for photocatalytic reaction.

Based on the combined experimental results and DFT analyses, we illustrate the schematic diagrams to elucidate the potential photocatalytic mechanism from viewpoints of structure, and directions of charge separation and transportation in **Figure** **8**. Assuming that the staggered alignment band structure is constructed between ZIS and CS (Figure 8A), the CB electrons of ZIS can transfer to CB of CS, meanwhile the VB holes of CS could migrate to the VB of ZIS with the help of the internal electric field (IEF). In such a case, the VB holes of ZIS could not have the ability to oxide H_2_O to •OH, as evidenced by the in situ EPR observations. Moreover, XPS shift analysis combined with DFT calculations confirm that the electrons transfer happens from CS to ZIS, leading to the formation of Z‐scheme photocatalytic system (Figure 8B). Under visible light (*λ* ≥ 420 nm) irradiation, the electrons are excited from VB to the CB for both CS and ZIS. Thus the photogenerated electrons in the CB of CS can move to the VB of ZIS and recombine with the photogenerated holes, which simultaneously reserve a more negative CB potential of ZIS and a more positive VB potential of CS for the reduction and oxidation of reactants, respectively. In such a case, the lifetime of electrons on the CB of ZIS is prolonged, thereby leading to an efficient water reduction reaction. Meanwhile, the holes in the VB of CS can be oxidized by the sacrificial electron donor S^2‒^/SO_3_^2‒^, which remarkably suppresses the backward reaction and enhances the charge separation efficiency. From the architecture point of view (Figure 8C), constructing the cube‐in‐cube ZIS/CS photocatalyst indeed provides abundant active sites, benefits for light harvesting as well as shortens the charge transfer distance to the surface due to the hollow nature of CdS and thin nanosheets of ZnIn_2_S_4_.

## 3 Conclusion

In summary, we have constructed the hierarchical ZnIn_2_S_4_/CdS nanostructures via a multistep synthesis route, in which ZnIn_2_S_4_ ultrathin nanosheets were uniformly grown on surface of hollow CdS. Experimental observations and DFT calculations demonstrate that the photogenerated electrons transfer from CdS to ZnIn_2_S_4_, thereby confirming the Z‐scheme heterojunction configuration. Detailed mechanism investigation unveils the pivotal role of this unique Z‐scheme photocatalytic system resulting in facilitated charge separation and transfer, abundant reaction active sites, good light harvesting ability, and shortened charge transfer distance to the surface, and thus dramatically enhanced the photocatalytic H_2_ generation activity. Compared to bare CdS, the optimized ZnIn_2_S_4_/CdS exhibits a remarkable H_2_ generation rate of 7.4 mmol g^−1^ h^−1^ under visible‐light irradiation (*λ* ≥ 420 nm), high AQE value of 12.6% at 420 nm as well as good photochemical stability. This work helps to in‐depth understand the good charge utilization and solar light harvesting for achieving highly efficient photocatalytic activity.

## 4 Experimental Section

### Synthesis of Cd‐PBAs Precursor

The Cd‐PBAs (Prussian blue analogs) precursor was prepared by a mild coordination reaction at room temperature. More specifically, 0.6 mmol of CdCl_2_·2.5H_2_O, 1.00 g of PVP, and 0.35 mmol of C_6_H_5_Na_3_O_7_·2H_2_O were dissolved in 20 mL of H_2_O to form solution A. Then, solution B was prepared by dissolving 0.4 mmol of K_3_[Co(CN)_6_] in another 20 mL of H_2_O and dropwise added it to solution A. After aging for 1 h, the obtained products were collected and washed with ethanol, and added into 100 mL of mixture solution (v/v of deionized water and ethanol, 1/1). Last, solution C was achieved.

### Synthesis of Hollow CdS Cube

Hollow CdS cube was obtained by the sulfurization process at room temperature. Generally, 0.200 g of TAA and 0.8 mL of NH_3_·H_2_O was orderly added in solution C with gentle stirring. After reaction for 1.5 h, 1 mL of 100 mM Na_2_S solution was added. After aging for 30 min, the obtained products were collected and washed with ethanol, and added into 100 mL of mixture solution (v/v of deionized water and ethanol, 1/1).

### Synthesis of Hierarchical ZnIn_2_S_4_/CdS and ZnIn_2_S_4_

Hierarchical ZnIn_2_S_4_/CdS composite was fabricated via a hydrothermal reaction. Typically, for synthesis of ZnIn_2_S_4_/CdS with 30 mol% of ZnIn_2_S_4_ (labeled 30% ZIS/CS), 90 mL of CdS intermediate solution, 0.540 g of InCl_3_·4H_2_O, 0.272 g of ZnCl_2_ and 0.300 g of TAA were dissolved in 80 mL of HCl aqueous solution (pH = 2.5) with stirring. Then, 20 mL of glycerol was added to the mixture and reacted at 80 °C for 2 h. After that, the target product 30% ZIS/CS was collected by centrifugation and washed with ethanol. For the synthesis of *x*% ZIS/CS (*x* = 10, 20, 40 and 50), the volumes of CdS cube solution were 30, 60, 120 and 150 mL, respectively. For comparison, pristine ZnIn_2_S_4_ was also synthesized by a similar procedure to that of ZnIn_2_S_4_/CdS without adding CdS.

### DFT Calculations

To analyze the interfacial effect of ZnIn_2_S_4_/CdS heterojunction, Python was used to build the ZIS(001)/CS(101) interface model with ≈15 Å thickness, which contains 137 atoms. To study the interface character of ZIS/CS heterojunction, the constructed model was not stoichiometric. The DFT calculations were performed via VASP using the projector augmented wave potential from the website (https://materialsproject.org/materials/). Perdew–Burke–Ernzerhof functional with LDA+U density functional approach was adopted. To accurately obtain the density of electronic states, the plane wave cutoff energy was 400 eV and the energy convergence was 1 × 10^−4^ eV. During the relaxation, part atoms in ZIS were fixed and the atomic positions at the heterojunction were optimized. The Hellmann–Feynman forces convergence criterion was set as less than 0.05 eV Å^‒1^. The electronic energy was considered self‐consistent when the energy change was smaller than 10^−5^ eV. The K‐point of 3 × 3 × 1 was used for the optimization of the ZIS (001) facet and CS (101) facet, and the thicknesses ≈12.0–13.0 Å was anchored. The constructed heterojunction was used for the calculation of charge density difference, electrostatic potential, work function (WF), and density of states (DOSs). The charge density difference and WF can be obtained after the convergence of self‐consistent calculation. When self‐consistent structure was used as the input file, the wave function and charge density files can be read to obtain the state density of the heterojunction.

## Conflict of Interest

The authors declare no conflict of interest.

## Supporting information

Supporting Information

## Data Availability

Research data are not shared.
