# Supplementary material for: Constructing Direct Z‐Scheme Heterostructure by Enwrapping ZnIn2S4 on CdS Hollow Cube for Efficient Photocatalytic H2 Generation
Source: Adv Sci (Weinh). 2022 Jun 24;9(24):2201773. doi: 10.1002/advs.202201773 (PMC9404389; doi:10.1002/advs.202201773)
Supplement: Supplementary file 1 — Supporting Information [file ADVS-9-2201773-s001.pdf]

ADVANCED SCIENCE
Open Access

Supporting Information

for *Adv. Sci.*, DOI 10.1002/advs.202201773

Constructing Direct Z-Scheme Heterostructure by Enwrapping $ZnIn_2S_4$ on CdS Hollow Cube for Efficient Photocatalytic H_2 Generation

Chuan-Qi Li, Xin Du, Shan Jiang, Yan Liu, Zhu-Lin Niu, Zhong-Yi Liu, Sha-Sha Yi and Xin-Zheng Yue**

Supporting Information

Constructing Direct Z-Scheme Heterostructure by enwrapping $ZnIn_2S_4$ on CdS Hollow Cube for Efficient Photocatalytic H_2 Generation

Chuan-Qi Li, Xin Du, Shan Jiang, Yan Liu, Zhu-Lin Niu, Zhong-Yi Liu, Sha-Sha Yi, and Xin-Zheng Yue**

[a]College of Chemistry, Zhengzhou University, Zhengzhou 450001, China.

[b]Henan Institutes of Advanced Technology, Zhengzhou University, Zhengzhou 450003, China.

[c]School of Materials Science and Engineering, Zhengzhou University, Zhengzhou 450001, China.

***Corresponding author:** yiss@zzu.edu.cn; yuexz@zzu.edu.cn

1. Experimental section

1.1. Chemicals and materials

Polyvinylpyrrolidone (PVP, Mw = 1.3 × 10^6), cadmium chloride hemi-pentahydrate ($CdCl_2 \cdot 2.5H_2O$, 98%), sodium citrate ($C_6H_5Na_3O_7 \cdot 2H_2O$, 99%), potassium hexacyanocobaltate ($K_3[Co(CN)_6]$, 98%), thioacetamide (TAA, ≥99%), sodium sulfide (Na_2S, 95%) and sodium sulfite (Na_2SO_3, 98%) were purchased from Aladdin. Ethanol (CH_3CH_2OH, ≥95%), ammonium hydroxide ($NH_3 \cdot H_2O$, 25%~28%), potassium permanganate ($KMnO_4$, ≥99.5%) and hydrochloric acid (HCl, 36%~38%) were bought from Shanghai Chemical Reagent Co., Ltd. Glycerol ($HOCH_2CHOHCH_2OH$, ≥99%) was purchased from Tianjin Fengchuan Chemical Reagent Technology Co., Ltd. Fluorine-doped tin oxide ($F:SnO_2$, FTO, <15 ohm sq^{-1}) glass was purchased from Zhuhai Kaivo Optoelectronic Technology Co., Ltd. Deionized water (18.2 MΩ·cm) was prepared in the laboratory by instrument SZ-93A automatic pure water distiller (Shanghai Yarong biochemistry instrument factory). Zinc nitrate hexahydrate ($Zn(NO_3)_2 \cdot 6H_2O$, 99%) and 2-methylimidazole ($C_4H_6N_2$, 98%) were purchased from Aladdin. Methanol (CH_3OH, 99.5%) was bought from Tianjin Kermel Chemical Reagent Co., Ltd. Ammonium Perrhenate (NH_4ReO_4, 99.99%) was purchased from Shanghai D&B Biological Science and Technology Co., Ltd. Sulfuric acid (H_2SO_4, 95%~98%) was bought from Luoyang Chemical Reagent Plant.

2. Characterization

Morphologies and microstructures of as-prepared catalysts were conducted by focused ion beam scanning electron microscope (FIB-SEM, Zeiss/auriga-bu), transmission electron microscopy (TEM)

and high-resolution TEM (HRTEM, FEI TalosF200S). X-ray diffraction (XRD) measurements were performed with a Rigaku D/Max-2550 diffractometer using Cu Kα radiation (λ = 1.54056 Å) at 50 kV and 200 mA in the 2θ range of 20-80° at a scanning rate of 4° min^{-1}. Electron paramagnetic resonance (EPR) spectra were performed on a Bruker A300 spectrometer to detect the hydroxyl radical (•OH) with the assistance of 5,5-Dimethyl-l-pyrroline N-oxide (DMPO) under visible-light irradiation at room temperature. X-ray photoelectron spectroscopy (XPS) measurements were performed on an AXIS Supra spectrometer using monochromatized Al Kα excitation. Physisorption analyzer (Micromeritics ASAP 2420) was used to measure the surface area and the pore volume of the catalysts at −196°C. Before each measurement, the samples were degassed at 150°C for 3 h to remove the physically adsorbed moisture. The UV-vis diffuse reflectance spectra (UV-vis DRS) were measured on a UV-vis-NIR spectrophotometer (Shimadzu UV-3600) equipped with an integrating sphere assembly, using $BaSO_4$ as the reflectance sample. The steady-state photoluminescence (PL) emission spectra with an excitation wavelength of 350 nm were measured on a spectrofluorometer (FluoroMax-4, HORIBA, France). A spectrofluorometer (FLS980, Edinburgh, England) was employed to record time-resolved PL (TRPL) spectra. The average lifetime (τ) of charge carriers can be estimated according to the following Equation S1:

$$\tau = \frac{\tau_1^2 A_1 + \tau_2^2 A_2}{\tau_1 A_1 + \tau_2 A_2} \qquad \text{(Equation S1)}$$

where τ_1 is the short carrier lifetime attributed to quasi-free excitons, τ_2 is the long component due to localized exciton recombination, A_1 and A_2 are the percentages of the short and long component in the total lifetime, respectively.

The lock-in-based surface photovoltage (SPV) spectrum was measured in a photovoltaic cell with a fluorine-doped tin oxide (FTO)-sample-FTO sandwich structure. The test system includes a 500 W Xe lamp (CHF-XM-500 W, Global Xenon Lamp Power) with a grating monochromator (Omni-5007, Zolix) as the monochromatic light source, a lock-in amplifier (SR830-D SP) and a light chopper (SR540). The light chopping frequency is 24 Hz. Before SPV test, the system was calibrated by a DSI200 UV enhanced silicon detector to eliminate the possible phase shift, which is not correlated to the SPV response. If so any phase retardation can reflect the kinetics of SPV response. Transient photovoltage (TPV) measurements were conducted under a laser radiation pulse with the wavelength of 355 nm and pulse width of 5 ns from a third-harmonic Nd:YAG laser (Polaris II, New Wave128 Research, Inc.). The intensity of the pulse was controlled with a neutral grey filter and determined with a Joule meter (Starlite, Ophir, Inc.). The transient signal was registered using a 500 MHz digital phosphor oscilloscope (TDS 5054, Tektronix).

3. Electrochemical and photoelectrochemical measurements

The electrochemical and photoelectrochemical (PEC) measurements were performed on an electrochemical workstation (RST5000, Shiruisi Instrument Technology Co., Ltd., Zhengzhou) using a three-electrode system at room temperature with 0.5 M Na_2SO_4 aqueous solution as the electrolyte. The as-prepared electrode film, Pt wire and Ag/AgCl (saturated KCl) were served as the working electrode, counter electrode and reference electrode, respectively. For PEC test, the simulated solar light was supported by a 300 W Xe lamp (CEL-HXF300, Beijing China Education Au-light Co., Ltd) equipped with an AM 1.5G optical filter. Electrochemical impedance spectroscopy (EIS) was conducted under light irradiation under open circuit voltage with an amplitude of 5 mV over the

frequency range from 100 kHz to 0.01 Hz. Open circuit potential (OCP) decay curves were employed to analyze the charge decay behaviors, which were performed after turning off the light irradiation. The carrier recombination rate can be fitted to the following Equation S2:

$$\frac{V - V_{light}}{V_{dark} - V_{light}} = 1 - \exp(-kt) \qquad \text{(Equation S2)}$$

where V, V_{light} and V_{dark} are the OCP values (V) at any time, under irradiation and in dark, respectively. k is the pseudo-first order recombination rate constant (s^{-1}). The potential dependent carrier lifetime determined from OCP decay of the accumulated electrons can be measured in qualitative using the following Equation S3:

$$\tau_n = \frac{k_B T}{e}\left(\frac{dV}{dt}\right)^{-1} \qquad \text{(Equation S3)}$$

where k_B is the Boltzmann constant (1.38×10^{-23} J K^{-1}), T is the absolute temperature (298 K), e is the charge of electron (1.602×10^{-19} C), and dV/dt is the derivative of the OCP transient decay. Current-time (*i*-*t*) curves were conducted with a light on/off cycle for every 40 s at 0 V *vs*. Ag/AgCl. Mott-Schottky (M-S) plots were performed and recorded over an AC frequency of 1000, 2000 and 3000 Hz in dark condition to obtain the flat band potentials (E_{fb}) according to the following Equation S4:

$$\frac{1}{C^2} = \frac{2}{e\varepsilon\varepsilon_r N_D}\left[\left(E - E_{fb}\right) - \frac{\kappa T}{e}\right] \qquad \text{(Equation S4)}$$

where C is the space charge capacitance (F cm^{-2}), ε_r is the relative dielectric constant of CdS or $ZnIn_2S_4$, ε is the vacuum permittivity (8.85×10^{-12} F m^{-1}), N_D is the charge donor density (cm^{-3}), E is the electrode applied potential (V), κ is the Boltzmann's constant (1.38×10^{-23} J K^{-1}), T is the absolute temperature (K) which is generally small and could be neglected, and e is the electron

charge (1.60×10^{-19} C).

4. Photocatalytic H_2 generation

The photocatalytic water splitting experiments were performed in a quartz glass reaction cell connected to an automatic on-line trace gas analysis system (Labsolar 6A, Beijing Perfectlight). In a typical experiment, 25 mg of photocatalyst was dispersed in a 0.35 M Na_2S + 0.25 M Na_2SO_3 aqueous solution (100 mL). After the system was degassed completely, the reaction solution was irradiated by a 300 W Xe lamp (PLS-SXE300D, Beijing Perfectlight) equipped with a 420 nm cut-off filter. The photoreactor was equipped with a cooling circulating water flow to maintain a constant temperature (5°C). H_2 gas generation was analyzed using an online gas chromatograph (GC-2014C, Shimadzu Co., Japan, with Ar as the carrier gas) equipped with an MS-5A column and a thermal conductivity detector (TCD). The stability tests were performed for 4 cycles of 20 h. After every cycle was done, the system needs to be fully degassed until vacuumed.

The apparent quantum efficiency (AQE) was measured under monochromatic light irradiation with the band-pass filters of 420, 450 and 500 nm, respectively. In detail, 25 mg of photocatalyst was dispersed in 100 mL of 0.35 M Na_2S + 0.25 M Na_2SO_3 aqueous solution. After the system was degassed completely under stirring, the solution was irradiated by a 300 W Xe lamp equipped with a band-pass filter. The AQE was calculated from the following formula:

$$\text{AQE} = \frac{2N1}{N2} \times 100\%$$

where *N1* and *N2* represent the number of evolved H_2 molecules and number of incident photons, respectively.

$$\mathrm{N1} = \mathrm{H_2\ amounts\ evoluted} \times \mathrm{Avogadro's\ Constant}$$

$$\mathrm{N2} = \frac{E\lambda}{hc}$$

where Avogadro's constant is 6.02×10^{23} molecules mol^{-1}, E is the power of lamp source (W m^{-2}), λ is the wavelength of the monochromatic light (nm), h is the Planck's constant (6.63×10^{-34} J s), c is the speed of light (3.0×10^{8} m s^{-1}), and the surface area of incident light is 40.69 cm^2. For monochromatic light of 420 nm, the light intensity is read as 44.76 W m^{-2}.

2. Supplemental Figures and Tables

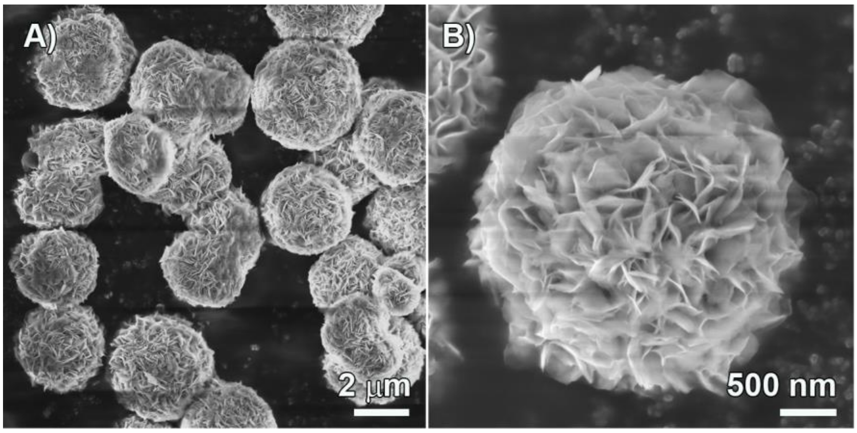

Figure S1. (A) low- and (b) high-resolution FIB-SEM images of bare $ZnIn_2S_4$ nanoflowers.

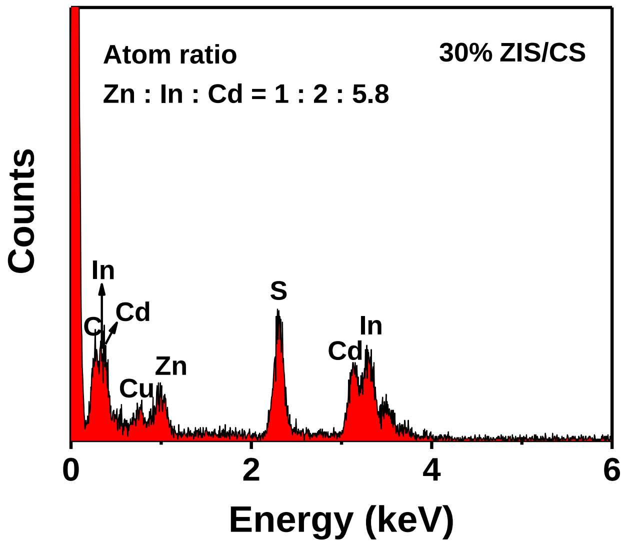

Figure S2. EDX spectrum of 30% ZIS/CS.

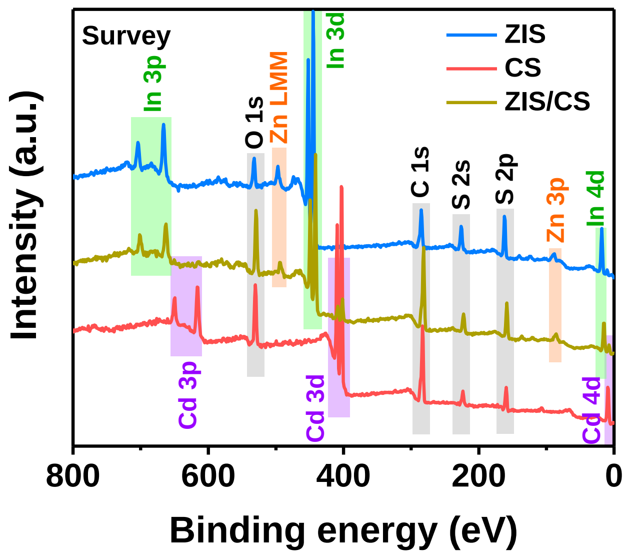

Figure S3. Survey XPS spectra of ZIS, CS and ZIS/CS.

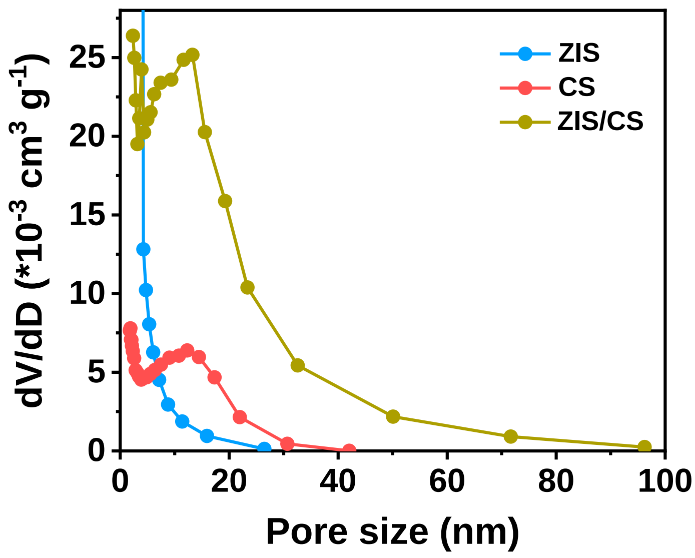

Figure S4. Pore size distributions of ZIS, CS and ZIS/CS.

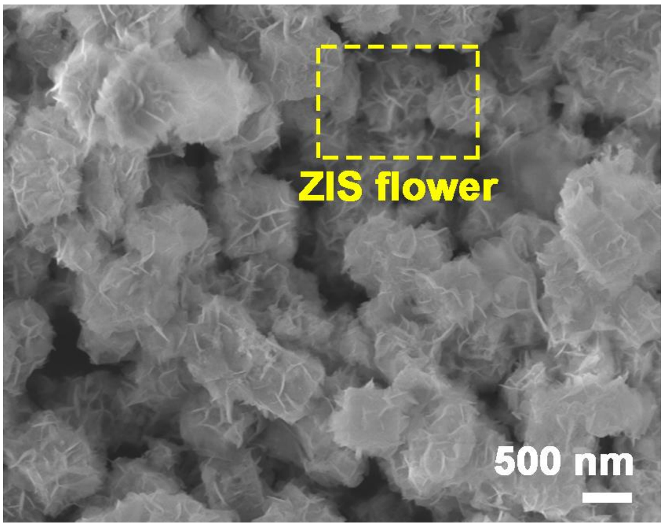

Figure S5. FIB-SEM image of 50% ZIS/CS.

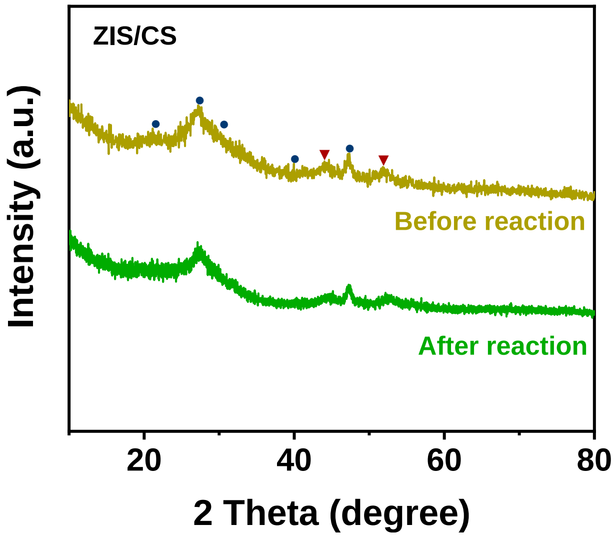

Figure S6. XRD patterns of ZIS/CS before and after the long-term photocatalytic H_2 generation reaction.

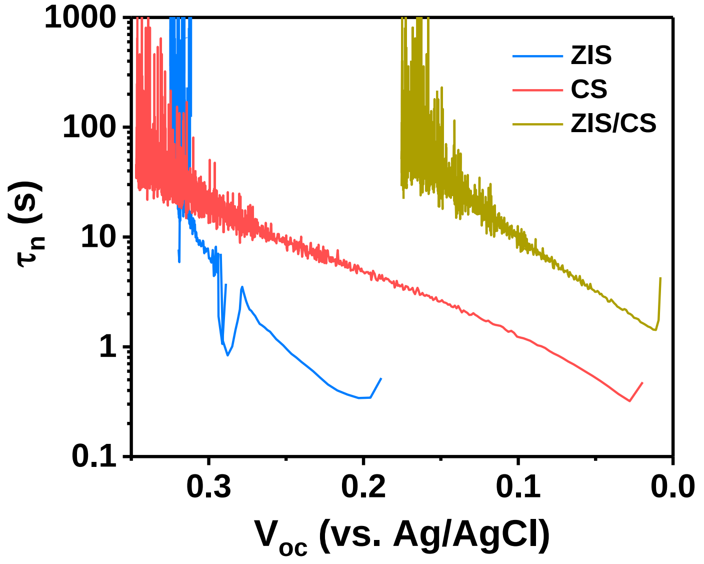

Figure S7. Potential dependent carrier lifetime determined from OCP decay in dark for ZIS, CS and ZIS/CS.

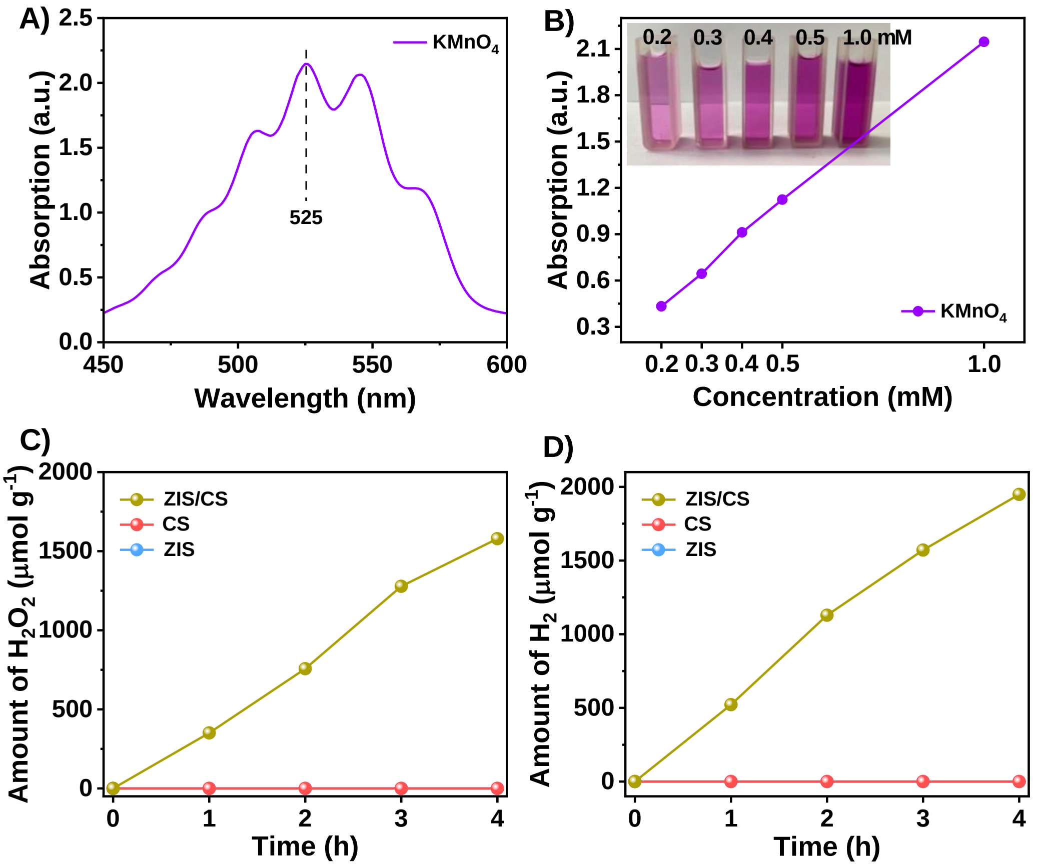

Figure S8. (A) UV-vis absorption of 1.0 mM $KMnO_4$ solution. (B) Absorption intensity of $KMnO_4$ solution at 525 nm as a function of the solution concentration; inset is the pictures of $KMnO_4$ solution at different concentrations. Time course of (C) H_2O_2 and (D) H_2 genenration over CS, ZIS and ZIS/CS in pure water under visible light irradiation ($\lambda \geq 420$ nm).

Figure S8 shows the photocatalytic activities of ZIS, CS and ZIS/CS in pure water under visible light irradiation ($\lambda \geq$ 420 nm). No O_2 generation was detected during the whole reaction process. To estimate whether there are H_2O_2 generated and its amount evaluated, the redox titration with $KMnO_4$ was performed ($2MnO_4^- + 5H_2O_2 + 6H^+ \rightarrow 2Mn^{2+} + 5O_2 \uparrow + 8H_2O$). Figure S8A shows the UV-vis absorption of 1.0 mM $KMnO_4$ solution, illustrating the absorption peak at 525 nm. During the photocatalytic reaction of ZIS/CS, 1 mL of solution was taken from the reaction system at interval of 1 h to react with $KMnO_4$ solution (0.5 mM, 2 mL). The H_2O_2 generation amount was

detected according to the concentration dependent absorption intensity of $KMnO_4$ solution (Figure S8B).

Figure S8C,D show the time course of H_2O_2 and H_2 generation over CS, ZIS and ZIS/CS in pure water. It is clear that both H_2O_2 and H_2 can be generated by optimized ZIS/CS, while pure CdS and $ZnIn_2S_4$ show no photocatalytic activities in pure water. The successful detection of H_2O_2 reveals the quite positive VB potential of ZIS/CS because of the high redox potential of H_2O_2/H_2O (1.78 V *vs.* NHE).[S1] These results indicate that the VB-holes of CdS could oxide H_2O to H_2O_2, thus proving the Z-scheme charge transfer pathway in ZIS/CS heterojunction.

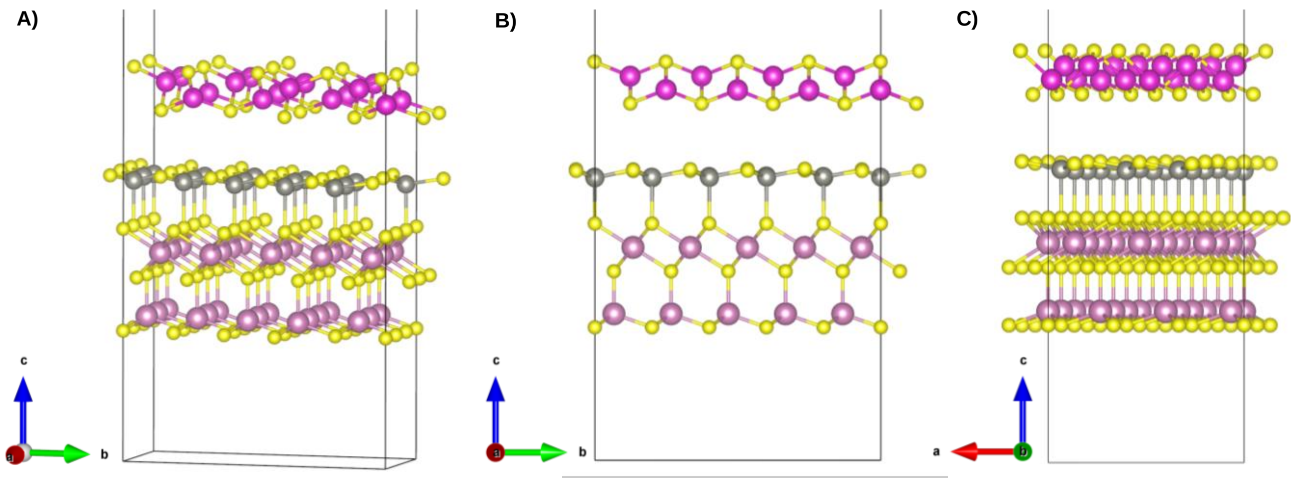

Figure S9. (A) Optimized structure diagram of ZIS/CS model for DFT calculations. (B) Front-view and (C) side-view of ZIS/CS model.

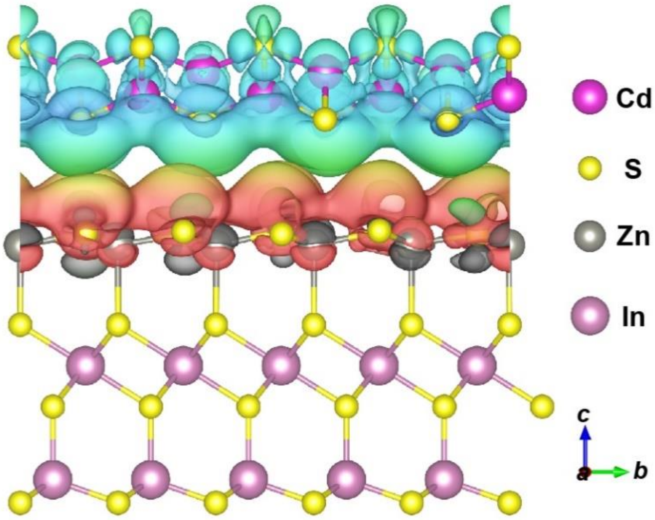

Figure S10. Calculated electrostatic potential of ZIS(001)/CS(101) interface.

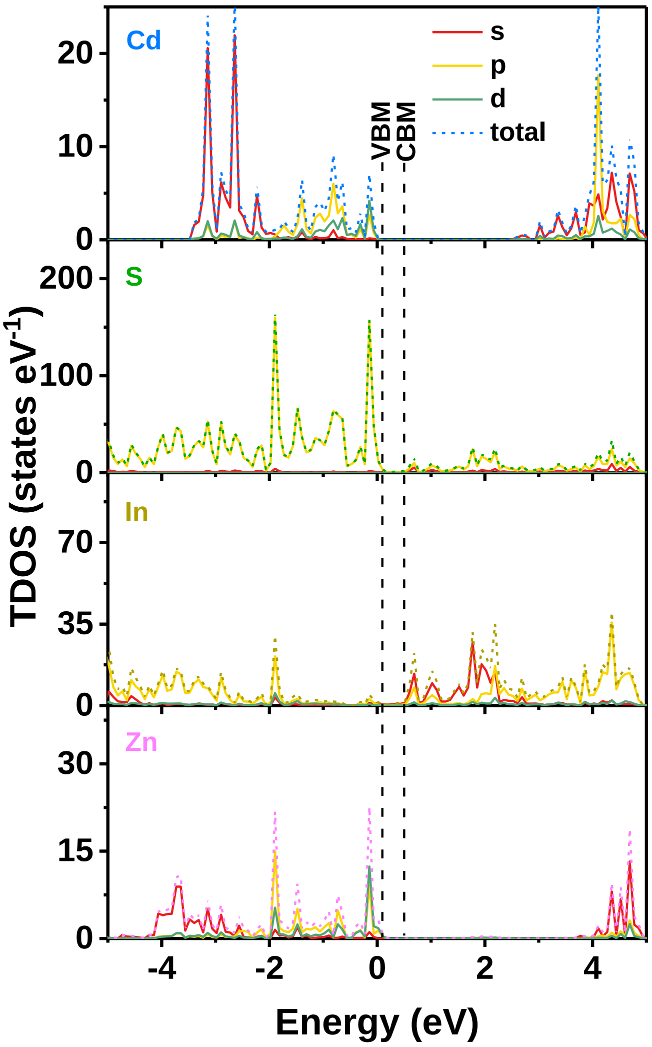

Figure S11. Calculated partial density of states (PDOSs) of Zn, In, S and Cd elements.

Figure S11 displays the partial density of states (PDOSs) of Zn, In, S and Cd elements and total density of states (TDOS) of ZIS/CS. From Figure S11, the following two key informations can be obtained: i) the orbital contribution of each element and ii) the conduction band maximum (CBM) and valence band maximum (VBM) constitutions. More specifically, Cd and S are at the VBM while the In and S are at the CBM, indicating the VB and CB of ZIS/CS composite are CdS and $ZnIn_2S_4$, respectively. In this way, the photocatalytic H_2 generation reaction was confirmed from the CB side of ZIS and the heterojunction interface realized the Z-scheme charge transfer path.

Table S1. Energy band structures of ZIS and CS.

**Sample**	**E_g (eV)**	**E_{CB} *vs.* NHE (V)**	**E_{VB} *vs.* NHE (V)**
ZIS	2.58	–1.05	1.53
CS	2.31	–0.46	1.85

Table S2. Dynamic analysis of emission decay for ZIS, CS and ZIS/CS.

**Sample**	**τ_1 (ns)**	**A_1 (%)**	**τ_2 (ns)**	**A_2 (%)**	**τ (ns)**
ZIS	1.5476	88.13	5.9198	11.87	3.0338
CS	1.5218	88.53	5.8901	11.47	2.9805
ZIS/CS	1.6497	90.52	7.1148	9.48	3.3505

References

[S1] J. Zhang, J. Lang, Y. Wei, Q. Zheng, L. Liu, Y.-H. Hu, B. Zhou, C. Yuan, M. Long, Efficient photocatalytic H_2O_2 production from oxygen and pure water over graphitic carbon nitride decorated by oxidative red phosphorus. *Appl. Catal. B Environ.* **2021**, *298*, 120522.
